# Efficacy of an Automated Pulmonary Embolism (PE) Detection Algorithm on Routine Contrast-Enhanced Chest CT Imaging for Non-PE Studies

**DOI:** 10.1007/s10278-025-01552-0

**Published:** 2025-06-25

**Authors:** Hayden R. Troutt, Kenneth N. Huynh, Aditya Joshi, Justin Ling, Scott Refugio, Scott Cramer, Jasmine Lopez, Katherine Wei, Amir Imanzadeh, Daniel S. Chow

**Affiliations:** UCI Health, Irvine, CA USA

**Keywords:** Pulmonary embolism, Artificial intelligence, Chest CT imaging, Computed tomography pulmonary angiogram

## Abstract

The urgency to accelerate PE management and minimize patient risk has driven the development of artificial intelligence (AI) algorithms designed to provide a swift and accurate diagnosis in dedicated chest imaging (computed tomography pulmonary angiogram; CTPA) for suspected PE; however, the accuracy of AI algorithms in the detection of incidental PE in non-dedicated CT imaging studies remains unclear and untested. This study explores the potential for a commercial AI algorithm to identify incidental PE in non-dedicated contrast-enhanced CT chest imaging studies. The Viz PE algorithm was deployed to identify the presence of PE on 130 dedicated and 63 non-dedicated contrast-enhanced CT chest exams. The predictions for non-dedicated contrast-enhanced chest CT imaging studies were 90.48% accurate, with a sensitivity of 0.14 and specificity of 1.00. Our findings reflect that the Viz PE algorithm demonstrated an overall accuracy of 90.16%, with a specificity of 96% and a sensitivity of 41%. Although the high specificity is promising for ruling in PE, the low sensitivity highlights a limitation, as it indicates the algorithm may miss a substantial number of true-positive incidental PEs. This study demonstrates that commercial AI detection tools hold promise as integral support for detecting PE, particularly when there is a strong clinical indication for their use; however, current limitations in sensitivity, especially for incidental cases, underscore the need for ongoing radiologist oversight.

## Introduction

Pulmonary embolism (PE) is the third most common acute cardiovascular condition and is associated with increased mortality, causing an estimated 180,000 deaths in the USA each year [1]. Unfortunately, each year, an estimated 600,000 cases of incidental PE go undiagnosed. This is largely due to the nonspecific clinical presentation of PE, which often overlaps with symptoms of other comorbid conditions, making accurate diagnosis challenging [2, 3]. This issue is further exacerbated by the increase in mortality risk to patients who are met with delays in diagnosis and delivery of appropriate therapy [4]. The urgency to accelerate PE management and minimize patient risk has driven the development of artificial intelligence (AI) algorithms designed to provide a swift and accurate diagnosis in dedicated chest imaging (computed tomography pulmonary angiogram; CTPA), or imaging obtained for clinically suspected PE; however, the accuracy of AI algorithms in the detection of incidental PE in non-dedicated, or routine, CT imaging studies remains unclear and untested.

Incidental PE is a frequent finding on CT scans conducted for reasons other than suspected PE. A study conducted by Dobler et al. showed that incidental PE was identified in 5.7% of hospitalized patients, including 16.7% of those over the age of 80, 24% of moderately to severely injured trauma patients, 3.6% of oncology patients, and 1.1% of individuals undergoing coronary CT scans [5]. Diagnosing PE on non-dedicated CT scans is more challenging, as incidental PE typically presents with a lower mean embolus burden compared to cases of clinically suspected PE. Moreover, these scans are not specifically optimized for visualizing the pulmonary arteries, often being susceptible to motion artifacts and suboptimal pulmonary arterial contrast enhancement [6]. The diagnosis and treatment of incidental PE are crucial, as studies suggest that anticoagulation therapy significantly reduces the risk of recurrent venous thromboembolism and mortality. In a pooled analysis of 11 cohort studies in cancer patients with incidental PE, those who received anticoagulation therapy had a 6-month recurrent venous thromboembolism risk of approximately 6.2%, compared to 12% in untreated patients, while all-cause mortality ranged from 28 to 37% in treated patients versus 47% in untreated patients [5]. AI models have the potential to assist radiologists in the diagnosis of incidental PE to alleviate the burden of missed diagnoses and improve detection accuracy, especially in non-dedicated CT scans.

AI-assisted radiology workflows have been found to substantially facilitate end-to-end processing of PE diagnosis on CTPA and treatment. One study of a tertiary referral center deploying a PE detection tool reports expedited time-to-consult for the patient cohort that received the AI intervention, with an average time-to-consult of 240.45 min for the pre-AI cohort and an average time-to-consult of 6.72 min for the post-AI cohort [7]. Other models have demonstrated high specificity and sensitivity, achieving up to 93.5% and 86.6%, respectively, despite limited training datasets [8]. Similarly, the Viz PE commercial algorithm produced by Viz.ai (Tel Aviv, Israel) has been trained to identify PE, or venous thromboembolism (VTE), in dedicated CT imaging with 91% sensitivity and 92% specificity [9]. Comparable automated detection algorithms have improved PE treatment turnaround times and reduced the total length of hospital stay in patients diagnosed with PE [10].

Artificial intelligence systems have the potential to become a versatile tool for radiologists in determining the presence of pulmonary embolisms amidst nonspecific symptoms [11]. Currently, computer-aided diagnostic systems utilizing AI technology are capable of localizing acute PE in dedicated pulmonary artery CT Angiogram imaging studies, beyond solely identifying the presence of the abnormality [12]. This advanced feature of PE detection tools helps radiologists in the localization of the positive trigger, which can prevent dedicating unnecessary time and effort reviewing imaging in the event of a false positive [13]. Evidence to date suggests that AI models, while incapable of replacing radiologists, can reliably serve as a safety net for PE diagnoses [14]. However, literature evaluating the performance of commercial algorithms in detecting incidental PE in non-dedicated contrast-enhanced CT chest scans remains limited [15].

The purpose of this study is to fully explore the potential for the Viz PE automated AI detection algorithm to identify incidental PE in non-dedicated contrast-enhanced CT chest imaging studies. We hypothesize that the AI tool utilized for detecting PEs will exhibit high sensitivity and specificity in non-dedicated pulmonary embolism contrast-enhanced CT chest scans. This study aims to provide valuable insights into the potential utility of AI-driven pulmonary embolism detection in non-dedicated CT chest scans, facilitating more efficient and accurate diagnosis.

## Methods

This retrospective study was conducted at a tertiary care center and was self-determined as Non-Human Subject Research by the lead researcher. The sample imaging data for the study was sourced from the institution’s Data Steward**.** A total of 193 consecutive contrast-enhanced CT chest exams performed between July 2023 and August 2023 were retrospectively collected and de-identified. These imaging studies were used as the dataset for algorithm analysis and evaluation of median resident performance.

Viz PE is a commercial deep learning-based pulmonary embolism detection algorithm developed for contrast-enhanced CT pulmonary angiograms. While the proprietary nature of the software precludes disclosure of specific architectural parameters, training data sources, or hyperparameter settings, publicly available information indicates that the model has been trained and validated on multi-institutional datasets with diverse imaging characteristics. The software integrates with picture archiving and communication systems (PACS), enabling real-time analysis and flagging of suspected PE cases. For this study, de-identified DICOM images were batch uploaded to the Viz.ai platform and automated results were returned for each case without additional pre-processing or user intervention. No local fine-tuning or algorithm customization was performed.

This tool, originally developed to evaluate contrast-enhanced CT chest imaging clinically indicated for PE, was deployed to identify the presence of PE on both dedicated and non-dedicated contrast-enhanced CT chest exams. Of the 193 total imaging studies evaluated using the Viz PE commercial tool, 130 had a prior clinical indication for PE documented in their order summaries, whereas the remaining 63 exams did not (Table 1). Three radiology residents (identified as SC, KH, and SR) independently interpreted the collected imaging studies. Their interpretations were compared with that of the commercial AI PE Detection tool, serving as a reference standard for analysis.

**Table 1:** Dataset overview

Dataset overview	
Dataset size	193
PE studies	130
Non-PE studies	63
Median age (yrs)	62
Male (total)	84
Female (total)	109
Male (%)	43.52%
Female (%)	56.48%

To establish a reliable reference standard, each case was independently reviewed by two of the three radiology residents, all of whom were in advanced stages of training (R2–R4) with no prior exposure to AI interpretation tools. All residents adhered to a standardized diagnostic protocol that involved binary classification of PE presence or absence based solely on imaging findings. In the event of interpretation disagreement, observed in a single discordant case, a senior radiologist reviewed the imaging to adjudicate the final diagnosis. This workflow ensured consistency and minimized bias in the diagnostic standard used to evaluate AI model performance.

The interpretations made by radiology residents were compared with the outputs of the commercial AI PE Detection tool to analyze the tool’s performance. Specificity, sensitivity, and overall accuracy were stratified by the presence or absence of a clinical indication for PE in the order summary, allowing for a detailed analysis of the commercial AI tool’s efficacy across these subgroups.

The level of consistency or agreement between each pair of residents’ CT PE interpretations was measured using percent agreement and Cohen’s kappa coefficient. Using the resident interpretations as reference standards, the predictions made by the AI PE detection tool were categorized as true positives (TPs), false positives (FPs), true negatives (TNs), or false negatives (FNs). These values were used to calculate the overall sensitivity, specificity, and accuracy of the predictions. Furthermore, the AI tool’s performance metrics were grouped based on CT type for comparison: non-dedicated contrast-enhanced CT chest studies versus dedicated CT chest studies. This grouping allowed for a comprehensive comparison of the AI tool’s performance across various imaging types.

## Results

The 193 predictions made by the commercial AI PE detection tool were overall 90.16% accurate, with a sensitivity of 0.41 and a specificity of 0.96. There were 9 true positives (TPs), 6 false positives (FPs), 13 false negatives (FNs), and 165 true negatives (TNs) (Table 2.).

**Table 2:**
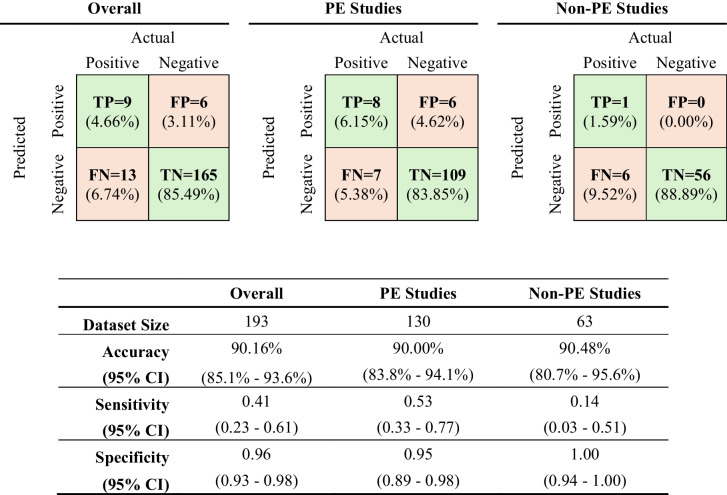
AI PE detection tool confusion matrix and performance metrics

**Fig. 1:**
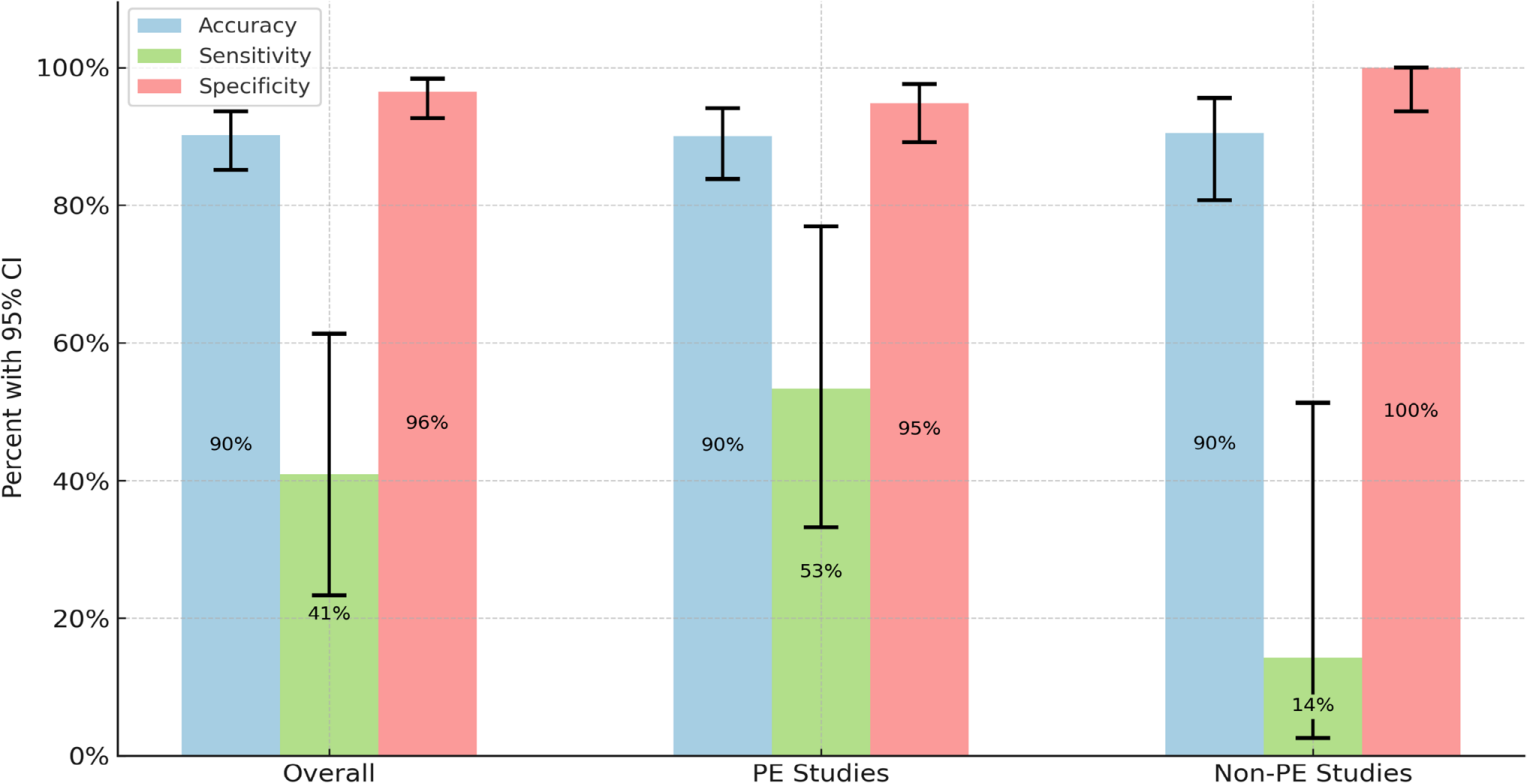
AI PE detection tool accuracy, sensitivity, and specificity

The predictions were categorized based on PE study type as well. The predictions for the 130 PE indicated contrast-enhanced chest CT imaging studies were 90.00% accurate, with a sensitivity of 0.53, and a specificity of 0.95. There were 8 TPs, 6 FPs, 7 FNs, and 109 TNs (Table 2.). The predictions for the 63 contrast-enhanced chest CT imaging studies without PE indication were 90.48% accurate, with a sensitivity of 0.14 and specificity of 1.00. There were 1 TP, 0 FPs, 6 FNs, and 56 TNs (Table 2.). The confidence intervals indicated in Fig. 1 and Table 2. were calculated using the Wilson score interval.

Most interpretations were consistent across residents, with only 2 cases of disagreement occurring between SC and KH. The percent agreement was 96.97% with a kappa coefficient of 0.88 between residents SC and KH. The percent agreement was 100% with a kappa coefficient of 1 between residents SC and SR, as well as KH and SR.

## Discussion

Pulmonary embolism (PE) remains a critical global health concern as it contributes to high rates of morbidity and mortality, necessitating prompt diagnosis to mitigate adverse outcomes [16]. The increasing use of contrast-enhanced imaging has allowed for more frequent incidental detection of PE, especially in patients undergoing routine CT examinations for oncologic, cardiovascular, and trauma-related evaluations [5, 17]. However, these incidental PEs are often overlooked as radiologists prioritize the primary clinical indication rather than secondary or incidental findings [18]. This oversight can result in severe outcomes, ranging from unnoticed progression to sudden fatal events. As the volume of non-dedicated CT increases, radiologists face interpretive challenges amidst heavy workloads, making automated detection tools valuable adjuncts for flagging potential PEs and enhancing detection consistency. This study explored the potential of an AI tool, specifically the Viz.ai PE detection algorithm, to assist radiologists in identifying PEs in non-dedicated contrast-enhanced CT chest imaging.

Non-dedicated contrast-enhanced CT chest studies differ substantially from dedicated CTPA protocols. Unlike dedicated CTPA protocols optimized for pulmonary artery visualization, non-dedicated CT chest studies, obtained during different phases of contrast enhancement, result in suboptimal visualization of PE. It has been reported that around 6% of CT scans done on hospitalized patients have incidental PE [5]. The potential value of automated PE detection algorithms is dependent on their ability to supplement radiologists’ efforts, flagging potential PEs for review, and improving detection consistency.

Our findings reflect that the AI PE detection tool demonstrated an overall accuracy of 90.16%, with a specificity of 96% and a sensitivity of 41%. Although the high specificity is promising for ruling out PE, the low sensitivity highlights a limitation, as it indicates the tool may miss a substantial number of true-positive incidental PEs. Notably, the sensitivity was higher for studies with a clinical indication for PE (53%) compared to those without (14%), underscoring the importance of pre-test probability and clinical context in driving automatic detection tool performance. The high specificity of the tool (95% for PE-indicated studies and 100% for non-PE-indicated studies) suggests it is highly effective in ruling in PE, which may contribute to a reduction in unnecessary follow-up tests. However, the tool’s limited sensitivity restricts its use as a standalone diagnostic tool.

The inter-reader agreement among radiology residents was also evaluated, which demonstrated consistency with Cohen’s kappa coefficients ranging from 0.88 to 1.00. This high level of agreement supports the reliability of resident interpretations as a reference standard. However, the perfect agreement between some residents raises concern for possible insufficient dataset variability. Future research should validate these findings using larger, more diverse datasets to determine whether the high agreement reflects genuine reliability or is an artifact of limited sample diversity.

To better understand the limitations of the AI model, we reviewed false-positive and false-negative cases across both PE-indicated and non-PE-indicated CT studies. The majority of false negatives were observed in non-dedicated contrast-enhanced chest CTs, where image quality was affected by motion artifacts. These artifacts likely impaired the model’s ability to accurately detect small or peripheral emboli. This is consistent with known challenges in AI-based chest imaging interpretation and highlights key areas for improvement. Future iterations of such algorithms could benefit from incorporating image quality scoring, artifact detection modules, or uncertainty quantification techniques to reduce diagnostic errors in heterogeneous imaging environments. Additionally, leveraging clinical data such as patient demographics, risk factors, and pre-test probability scores may provide valuable context and enhance diagnostic accuracy. These improvements would increase the detection of subtle incidental PEs.

Compared with prior studies, the specificity observed in our findings aligns with other AI models for PE detection, which typically report specificities between 93 and 95% in dedicated CTPA studies; however, the sensitivity observed here (41%) is lower than the typically reported range of 73–93% [8, 19–22]. Some studies have similarly leveraged advanced deep learning architectures for enhanced PE detection with significant diagnostic accuracy, even in challenging contexts such as COVID-19 patient populations [23]. This discrepancy in sensitivity performance likely stems from the limitations of non-dedicated imaging, including reduced image quality, lower embolus burden, and the absence of optimized protocols for pulmonary vasculature visualization. These factors hinder the model’s performance, particularly for subtle or incidental PEs.

Notable limitations of this study include the small sample size of CTPA exams used relative to the frequency of incident PE on routine imaging. In addition, the minimal demographic data collected from a single tertiary treatment center to support this study may impact the external validity of the findings. Further research could address these concerns and improve model generalizability by integrating larger and more diverse datasets, including more incidental PE cases, leveraging semi-supervising training techniques, and more efficient image annotation strategies. These strategies, in turn, would enhance the sensitivity of AI models for detecting PE in non-dedicated imaging and generalizability [24].

The clinical implications of this study are noteworthy, as the AI tool’s high specificity indicates its potential as a valuable adjunct for ruling in PE, particularly in patients with low clinical suspicion. By effectively identifying negative cases, the AI tool can assist in reducing the workload of radiologists, enabling them to concentrate on cases with a higher likelihood of being positive or have greater complexity. Incorporating AI into diagnostic workflows as a triage or prioritization tool may lead to more consistent detection rates and improved efficiency, without compromising patient safety. Triaging cases using AI has led to a significant reduction in reporting turnaround times, improving patient outcomes [25]. Specifically, AI-based systems can be integrated into PACS to automatically analyze non-dedicated CT scans and flag suspected PE cases for prioritized review by a radiologist. For example, tools like Aidoc’s FDA-cleared PE module scan chest CT images in the background and notify radiologists in real-time when emboli are detected [26]. This integration optimizes the allocation of radiology resources, ensuring that urgent cases are addressed promptly. Beyond enhancing radiologist efficiency, triage tools offer substantial advantages for patients. One study found a 2.07-day (95% C.I. 0.1–4.0) reduction in length of stay for patients diagnosed with PE when an AI triage system was used [10]. This reduction may be attributed to faster diagnosis and treatment, minimized delays in imaging and reporting, and more focused resource allocation for high-risk patients.

AI PE detection tools hold promise as integral support for detecting PEs, particularly when there is a strong clinical indication for their use. While their high specificity is advantageous for ruling in PE, the current limitations in sensitivity, especially for incidental cases, underscore the need for ongoing radiologist oversight. Future advancements should aim to improve AI sensitivity for safe integration into clinical workflows as a reliable complement to radiologist expertise.
